# Biomonitoring with bees and bee products: multielement profiles including technology-critical elements

**DOI:** 10.1007/s00706-025-03425-2

**Published:** 2026-01-12

**Authors:** Simone Trimmel, Michael Schober, Johanna A. Lube, Thomas C. Meisel, Thomas Prohaska, Johanna Irrgeher

**Affiliations:** Chair of General and Analytical Chemistry, Montanuniversität Leoben, Leoben, Austria

**Keywords:** Chemometrics, Ecology, Mass spectrometry, Metals, Rare-earth elements

## Abstract

Honeybees (*Apis mellifera*) and their products are valuable biomonitors of environmental pollution. This study reports the contents of 48 elements in samples of propolis, bees, pollen, honey, honeycomb, and wax from bee hives in Leoben, Austria. The samples were taken in three campaigns over a period of twelve months. After microwave-assisted acid digestion, the samples were measured with inductively coupled plasma tandem-mass spectrometry (ICP-MS/MS) using nitrous oxide (N_2_O) as reaction gas for analytes affected by spectral interferences. Matrix-specific patterns in multielement variations were explored with univariate and multivariate statistical analysis. Principal component analysis (PCA) and hierarchical cluster analysis (HCA) revealed four matrix clusters (propolis; bee; pollen; honey/honeycomb/wax), with element mass fractions generally decreasing in the order propolis > bee > pollen > wax ≈ honeycomb ≈ honey. Two analyte groups could be separated by their PCA loadings, one interpreted as predominantly particle-associated (Li, Al, V, Cr, Fe, Nb, Y, La, Ce, Sm, Eu, Gd, Tb, Dy, Ho, Ta, Sb, Bi, Pb) and the other as more soluble/bioavailable (Na, Mg, Ca, Mn, Co, Ni, Cu, Zn, Ga, As, Se, Rb, Sr, Mo, Cd, Ba, Tl). Chondrite-normalized patterns of rare-earth elements including yttrium (REY) showed smooth profiles across all matrices, suggesting geogenic sources and negligible fractionation from biotransformation. Only minor differences were observed between different matrices from the same location. The observed contrasts in REY patterns in honey samples from Austria, Belgium, and Russia support their suitability for tracking regional environmental conditions.

**Graphical abstract:**

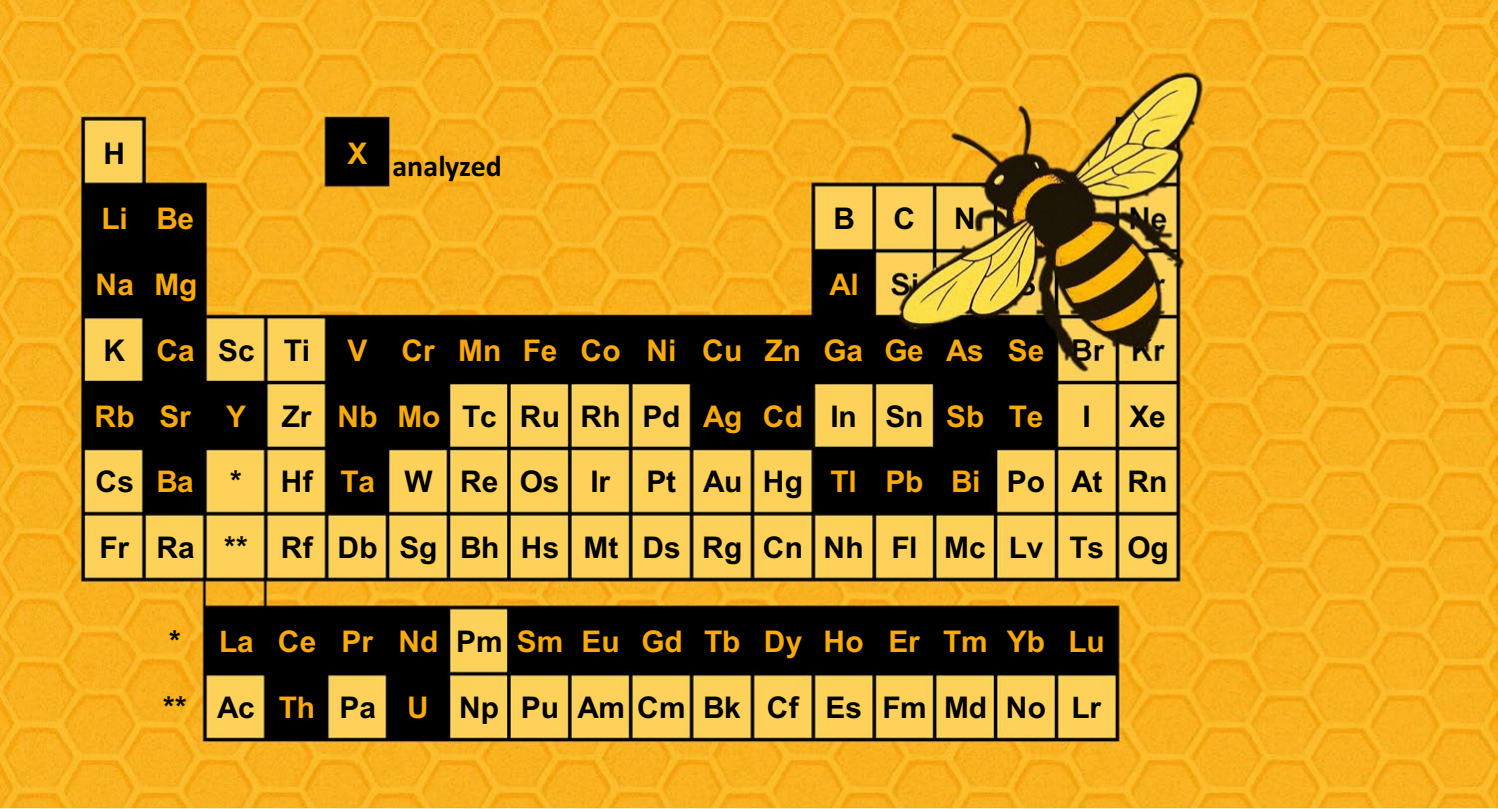

**Supplementary Information:**

The online version contains supplementary material available at 10.1007/s00706-025-03425-2.

## Introduction

Honeybees (*Apis mellifera*) are kept worldwide and exposed to environmental contaminants on their foraging flights, which cover an area on the order of a few square kilometers [1–3]. In addition to direct contact with atmospheric particulates, bees may ingest pollutants while collecting nectar and pollen [4, 5]. Consequently, honeybees and their products are widely established as biomonitors of environmental elemental deposition [6–10].

Among bee-related matrices, honey is by far the most extensively studied [7, 8, 11–19]. Its relevance extends beyond food safety to questions of authenticity and provenance. While the translocation of many potentially harmful elements such as Ni, Cd, and Pb from plants to honey is generally low even in moderately contaminated environments [7, 16, 20], the element content of honey is still routinely examined for risk assessment and testing of adulteration. Multiple studies have employed both elemental and isotopic signatures (e.g., *δ*^2^H/^1^H, *δ*^13^C/^12^C, *δ*^15^N/^14^N, *δ*^87^Sr/^86^Sr) to trace the floral and geographical origin of honey [6, 11, 14, 15, 21]. Furthermore, multielement compositions and isotope ratios have been successfully linked to those in water and in labile soil fractions (NH_4_NO_3_ extracts) [14].

The low levels of toxic elements in honey and in other metabolic bee products such as royal jelly [22] are attributed to transformation processes occurring in the bees’ metabolism. Pollutants absorbed with nectar are enzymatically converted into biocomplexes and subsequently either immobilized in the fat body or excreted with feces [23]. These processes explain the low transfer rates of contaminants into honey and royal jelly. At the same time, they suggest that matrices that undergo less biotransformation, such as pollen and propolis, may better reflect environmental pollution [24].

Table 1 provides a curated overview about the available literature data on the analytes and matrices investigated in the present study, emphasizing studies with underrepresented matrices and analytes to identify the current gaps in literature. In addition to forager bees, honey, and royal jelly, literature data on element contents is available for various other apicultural matrices, including drone bees [8], bee broods [8], beeswax [7, 8, 18], propolis [7, 18], bee bread [25], and pollen [7, 12, 18, 26].

**Table 1:** Curated (non-exhaustive) overview of literature data for the analytes and matrices investigated in the present study

Element	Propolis	Bee	Pollen	Wax	Honey
Li		[6, 9]			[14]
Be					[15, 16]
Na		[6, 8, 9]		[8]	[8, 11, 14, 17]
Mg	[18]	[6, 8]	[18]	[8, 18]	[8, 11, 13–15, 17–20]
Al		[6]	[26, 27]		[11, 13–16, 20]
Ca		[6, 8, 9]	[27]	[8]	[8, 13, 14, 16, 17, 19, 20]
V		[6]			[13–16]
Cr	[7, 28]	[1, 6–8]	[7, 26]	[7, 8]	[7, 8, 11, 13, 15–17]
Mn	[28]	[6, 8, 9]		[8]	[8, 11, 13–17, 19, 20]
Fe	[18, 28]	[6, 8, 9]	[18, 27]	[8, 18]	[8, 11, 13, 15–20]
Co		[6, 8]	[26]	[8]	[13–17]
Ni	[18, 28]	[8]	[18, 26, 27]	[8, 18]	[8, 11, 13–15, 17, 18, 20]
Cu	[29]	[6, 8]	[27]	[8]	[8, 11, 13–17, 19, 20, 29]
Zn	[18, 28, 29]	[6, 8]	[18]	[8, 18]	[8, 11, 13–16, 18, 20, 29]
Ga					[13, 14]
Ge					[16]
As	[28, 29]	[6, 8, 9]	[12, 26, 27]	[8]	[8, 12, 13, 15–17, 19, 29]
Se		[6]	[12]		[12–17, 20]
Rb		[6]			[11, 13–15]
Sr		[6]			[11, 13, 14, 16, 17]
Y			[12]		[12]
Nb					[12]
Mo		[6]			[14–17]
Ag					[17, 19]
Cd	[7, 18, 28, 29]	[1, 6–8]	[7, 12, 18, 26, 27]	[7, 8, 18]	[7, 8, 12, 13, 15–18, 20, 29]
Sb		[6]	[26]		[13, 15, 16]
Te					[13, 15]
Ba		[6, 9]	[26]		[11, 13–17]
La			[12]		[12, 14, 15, 19]
Ce			[12]		[12, 14, 15]
Pr			[12]		[12, 15]
Nd			[12]		[12, 14, 15]
Sm			[12]		[12, 14, 15]
Eu			[12]		[12, 14, 15]
Gd			[12]		[12, 15]
Tb					[15]
Dy			[12]		[12, 15]
Ho			[12]		[12, 15]
Er			[12]		[12, 15]
Tm			[12]		[12]
Yb			[12]		[12, 14, 15]
Lu			[12]		[12, 14, 15]
Ta					[12]
Tl		[6]			[13–17, 20]
Pb	[7, 18, 28, 29]	[1, 6–8]	[7, 12, 18, 26, 27]	[7, 8, 18]	[7, 8, 11–16, 18, 20, 29]
Bi			[12]		[12, 15]
Th					[15, 17]
U		[6]			[14–17]

So far, data on elements occurring at very low mass fractions remains limited, especially for emerging contaminant groups such as technology-critical elements (TCEs, e.g., Ga, Ge, Nb, Te, and the rare-earth elements (REYs, La-Lu and Y)). Altered environmental backgrounds of some of these elements have been linked to their increased use in modern materials and high-tech applications over recent decades [30–32], with potential ecotoxicological and human health impacts [33–37].

Given that bees integrate deposition from their entire foraging range and are closely associated with human-altered landscapes, apicultural matrices have promising potential for reflecting atmospheric deposition of TCEs. To explore this potential, the present study aims to expand the available data base on scarcely studied elements by a systematic study of the mass fractions of 48 elements (Li, Be, Na, Mg, Al, Ca, V, Cr, Mn, Fe, Co, Ni, Cu, Zn, Ga, Ge, As, Se, Rb, Sr, Nb, Mo, Ag, Cd, Sb, Te, Ba, Ta, Tl, Pb, Bi, Th, U, and the REYs) in European honeybees (*Apis mellifera*), propolis, pollen, wax, and polyfloral honey in a controlled environment in a suburban setting in Leoben, Austria. To test comparability of the obtained data, a limited number of honey samples from Russia and Belgium were additionally analyzed. This part of the study was conceived as a feasibility assessment, aiming to evaluate the applicability of the analytical approach and the potential for broader comparisons, rather than to provide statistically representative datasets for these regions. Absolute mass fractions, matrix-specific element patterns and REY fractionation were evaluated to assess their potential utility in biomonitoring.

## Results and discussion

The complete analytical dataset is given in the online supplementary information (SI-2, Tables A1–C2). Mass fractions of the analytes in all investigated samples are provided in Table A1, with repeatability expressed as relative standard deviations (RSDs) across 6 instrumental replicates per sample digest in Table A2. Limits of detection (*w*_L_) and limits of quantification (*w*_Q_) based on the procedural blanks are given in Table B1. Table B2 summarizes the mass fractions, biases and *z*-scores obtained for the in-house reference material based on sucrose and the certified reference material (CRM) GBW10015 Spinach Leaves (National Research Centre for Certified Reference Materials, China). For details, see section *Data processing*. The *p*-values of statistically significant observations (*p* < 0.05) in analyses of variances (ANOVAs) are given in Table C1.

### Elemental contents across apicultural matrices

Across all sampling campaigns, the elemental contents of the analyzed matrices revealed consistent patterns, with mass fractions generally decreasing in the order propolis > bee > pollen > wax ≈ honeycomb ≈ honey. Table 2 summarizes the mean mass fractions of selected scarcely analyzed TCEs across all sampling dates.

**Table 2:** Mean mass fractions *w* and intermediate precision expressed as standard deviations *s* in ng g^−1^ of Li, Be, Ga, Ge, Nb, Sb, Ta, Tl, and Bi in apicultural matrices from Leoben, Austria

		Propolis	Bee	Pollen	Wax	Honeycomb	Honey
Element	*n*	7	47	6	21	6	18
Li/ng g^−1^	*w*	66	24	43	14	< *w*_Q_	12
	*s*	17	14	30	10	n.a	15
Be/ng g^−1^	*w*	5.8	8.7	1.73	6.5	< *w*_Q_	0.66
	*s*	1.4	5.4	0.3	4.1	n.a	0.4
Ga/ng g^−1^	*w*	39	96	47	3.8	4.4	1.7
	*s*	10	37	13	2.8	1.7	1.4
Ge/ng g^−1^	*w*	10.9	3.4	2.48	2.3	< *w*_Q_	0.36
	*s*	3.3	1.8	0.99	n.a	n.a	0.29
Nb/ng g^−1^	*w*	54	5.4	12.8	2.8	1.18	0.25
	*s*	14	5.7	8.5	2.3	0.15	0.23
Sb/ng g^−1^	*w*	62	17	22	8.5	< *w*_Q_	5
	*s*	19	13	11	5.1	n.a	2.2
Ta/ng g^−1^	*w*	0.423	0.24	0.32	0.105	< *w*_Q_	0.063
	*s*	0.053	0.2	0.19	0.081	n.a	0.098
Tl/ng g^−1^	*w*	2.38	3.3	2.12	0.67	< *w*_Q_	0.47
	*s*	0.78	2.6	0.88	0.3	n.a	0.15
Bi/ng g^−1^	*w*	22.6	6.1	6.4	6.4	2	4.2
	*s*	7.7	6.6	2.1	6.9	1.6	7.9

*n* number of procedural replicates (independent digests) per matrix, < *w*_Q_ below limit of quantification, *s* n.a.: less than two replicates > *w*_Q_

**Fig. 1:**
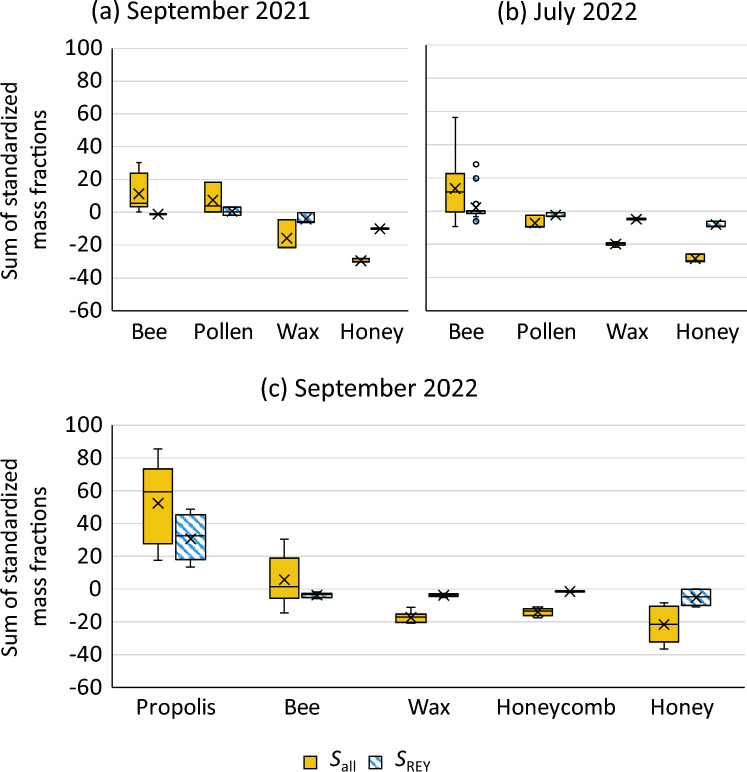
Sum of the standardized contents of all analytes (*S*_all_) and the REYs (*S*_REY_) by matrix and sampling campaign

Figure 1 illustrates the variation in the sum of standardized mass fractions across all elements (*S*_all_) and across all REYs (*S*_REY_). It is evident that *S*_all_ shows a decrease from bee—pollen—wax/honey in both cases where pollen samples were taken in correspondence to the excretion processes occurring in the bees’ metabolism. Propolis was only sampled in the last period and shows a significantly higher *S*_all_ than the other matrices. When considering individual elements, more differentiated patterns emerged, indicating element-specific accumulation or partitioning processes between the biological matrices.

In the September 2021 dataset (Fig. 1a), honeybees contained significantly higher mass fractions of Be, Na, Mg, Cu, and Zn than the other matrices. Within this campaign, *S*_all_ as well as the mass fractions of Ca, Ga, and Mo were also significantly higher in honeybees compared to wax and honey. Se mass fractions were significantly higher in honeybees than in pollen samples. Pollen, in turn, showed the highest Ca, V, and Nb mass fractions, and significantly higher Mg, Fe, Ni, and Cu than wax and honey. Al mass fractions in pollen exceeded those in both bees and honey, Sb was higher in pollen than in honey, and *S*_REY_ was significantly higher in pollen compared to wax.

In July 2022 (Fig. 1b), honeybees again showed the highest mass fractions of several elements, including Na, Mn, Co, Cu, Ga, and As. They also exhibited significantly higher levels of Mg, Ca, V, Fe, Rb, Sr, Mo, U, and *S*_all_ compared to honey and wax. Zn, Se, and Cd mass fractions in bees exceeded those in pollen and honey significantly. Ni, Ba, and *S*_REY_ were significantly higher in bees than in wax. Additionally, bees had significantly higher Pb contents than honey samples. Pollen samples contained elevated mass fractions of Mg, Ca, and Mo compared to honey and wax, and higher levels of Rb than wax.

In September 2022 (Fig. 1c), also propolis and honeycomb (wax with enclosed honey) samples were collected. Propolis contained the highest mass fractions of a range of elements, including Al, V, Cr, Fe, Nb, Sb, Pb, and Bi. It also showed the highest *S*_all_ and *S*_REY_ values. Honeybees showed highest mass fractions of Na, Mg, Ca, Mn, Cu, Zn, Ga, As, Rb, and Mo. Fe, Co, Sr, Tl, and *S*_all_ were significantly higher in bees than in honey, honeycomb, and wax. Al, Ni, Se, Cd, and Ta mass fractions were higher in bees than in honey and honeycomb.

Propolis, sampled only in September 2022, showed the highest mass fractions of a wide range of potentially toxic elements and TCEs. This may be attributed to its lipophilic nature, as it is composed largely of plant resins and waxes [38]. Moreover, its sticky surface [39] may enhance adhesion of fine atmospheric dust. The elevated levels of elements which are likely of the Earth’s upper crustal origin (e.g., Al, Cr, Fe) and anthropogenic tracers (e.g., Sb, Bi, Pb) found in the present study support previous conclusions that propolis can serve as a valuable biomonitoring matrix for airborne deposition, especially for toxic metals [24, 28, 29]. This matrix, which undergoes minimal biotransformation [39], may serve as a particularly sensitive indicator of atmospheric deposition of TCEs.

Overall, the relative differences among matrices in element mass fractions were broadly preserved across all sampling campaigns. In this study, honeybee bodies consistently contained elevated mass fractions of essential elements (e.g., Na, Mg, Ca, Mn, Zn). While their TCE contents were lower than those in propolis, they were higher than in the other investigated matrices. These elements may enter the bees through ingestion of nectar and pollen as well as deposit on their bodies through contact with airborne particles during foraging [40]. Thus, their elemental profile likely reflects a combination of external exposure (e.g., atmospheric deposition on the exoskeleton) and internalized intake via ingestion [41]. This reinforces their potential as a dynamic and sensitive biomonitor, especially if propolis samples are unavailable.

Pollen showed elevated levels of elements typically taken up by plants from the soil, such as Mg, Al, Ca, V, Fe, and Mo. This supports the interpretation that pollen reflects plant-mediated element uptake, which is influenced by both soil composition and atmospheric inputs. Although the time period is short, pollen is also exposed to air pollutants during the period it travels through air for pollination purpose [27]. Since bees collect pollen from multiple floral sources, it can serve as a broad integrator of elemental input from local vegetation. Yet, the findings of the present study strengthen the suggestion that pollen can be considered a representative bioindicator of environmental pollution to a lesser extent than honeybees [7].

Wax, honeycomb, and honey consistently exhibited the lowest mass fractions of most analytes, reflecting the metabolic transformation mechanisms described in previous studies [20, 23]. Although wax, honeycomb, and honey are low-accumulation matrices for elemental biomonitoring, honey analysis remains highly relevant for food-safety and authenticity monitoring. In the present study, honeycomb samples – defined as wax containing enclosed, unprocessed honey – displayed intermediate elemental mass fractions between pure honey and beeswax. This suggests that honey processing did not significantly alter the elemental profile of the enclosed matrix.

### Element partitioning by matrix

**Fig. 2:**
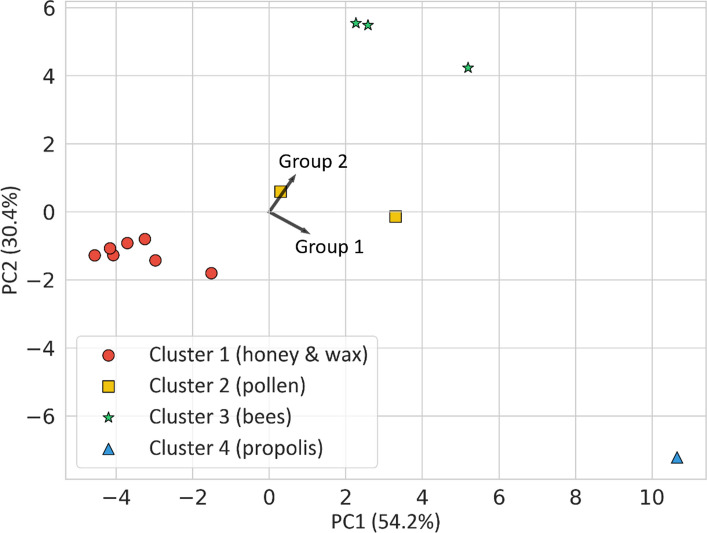
PCA biplot with colored symbols indicating matrix clusters. Black arrows represent average loading vectors for particulate-bound (group 1) and bioavailable (group 2) elements

The biplot of the principal component analysis (PCA) is shown in Fig. 2. Results from hierarchical cluster analysis (HCA) are visualized as overlays on the PCA biplot. PCA scores and cluster assignments of the individual samples are given in the SI-1, Table S1. The results revealed a clear separation of matrices along principal component (PC) 1 and 2, which collectively reflect the abundance and chemical characteristics of the analytes. Based on the angle of their directional loading vectors in the PCA space, the elements could be clearly separated into two major groups, indicating distinct co-variation patterns:

- Group 1 (lower right quadrant): Li, Al, V, Cr, Fe, Nb, Y, La, Ce, Sm, Eu, Gd, Tb, Dy, Ho, Ta, Sb, Bi, Pb
- Group 2 (upper right quadrant): Na, Mg, Ca, Mn, Co, Ni, Cu, Zn, Ga, As, Se, Rb, Sr, Mo, Cd, Ba, Tl

The PCA loadings and directional loading vector angles for each analyte are provided in the SI-1, Table S2. The separation between the two groups strongly aligns with element partitioning between dissolved and particulate phases in surface snow and river water reported in previous studies [42, 43]. Thus, the groups appear to show clear differences in chemical behavior and mobility.

Group 1 is dominated by elements commonly associated with particulate-bound or poorly soluble fractions in environmental matrices, with poor availability to plants, including several TCEs (Li, V, Nb, Ta, Sb, Bi, REYs), and elements with strong affinities to particles (Al, Cr, Fe). These elements are enriched in solid matrices that promote particle retention. In contrast, group 2 comprises elements which are more mobile in the dissolved phase and typically considered bioavailable or metabolically relevant, such as alkaline and alkaline-earth elements (Na, Mg, Ca, Rb, Sr, Ba) and nutrients such as Mn, Cu, and Zn.

This is in accordance with the patterns observed in univariate analysis: Bees clustered in the upper right quadrant (high PC1 and PC2), which aligns with group 2 and indicates an enrichment in soluble, physiologically incorporated elements. Propolis showed a distinct profile with high PC1 but negative PC2 scores, which points towards a dominance of group 1 elements. This is consistent with its resinous, particulate-rich nature. Honey, wax, and honeycomb all clustered on the upper side of the lower left quadrant. Their distance from both element vectors reflects their overall low element mass fractions. Their position suggests neither strong particulate nor bioavailable enrichment. Pollen samples, in turn, were located near the origin, suggesting mixed signatures and moderate elemental mass fractions.

### REY patterns

**Fig. 3:**
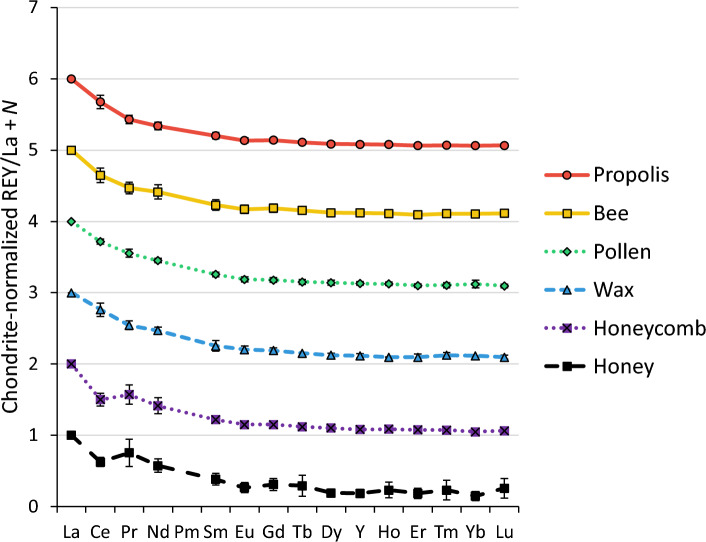
Chondrite- and La-normalized REY patterns. Error bars represent standard deviations of replicates (propolis: *n* = 7; bee: *n* = 16; pollen: *n* = 6; wax: *n* = 4; honeycomb: *n* = 3; honey: *n* = 8)

Figure 3 shows the mean chondrite-normalized REY patterns for each matrix. which are additionally normalized to La to allow for better comparison of their slopes. To visually distinguish the plots, a different integer offset *N* was added to each matrix.

As shown in Fig. 3, all matrices exhibit smooth REY patterns across the lanthanide series (La to Lu) including Y, with a slight but consistent negative Eu anomaly. Such patterns are typical for dust derived from the Earth’s upper crustal material [44, 45]. No matrix-specific fractionation in REY signatures is evident, aside from a slight Ce depletion in honeycomb and honey samples, likely caused by redox effects. This suggests that REYs undergo minimal biotransformation within the bees’ bodies. Given the predominantly particle-bound nature of REYs, this supports the interpretation that their presence in all the investigated apicultural matrices reflects a shared origin: dust particles of geogenic origin. These particles may adhere to plant surfaces or become attached to bees during foraging and in this way enter the hives.

The lack of matrix-specific REY fractionation is a promising finding for biomonitoring and the geographic provenance tracing of honey. It indicates that, unlike elements affected by metabolic processes, the REYs can serve as conservative tracers of dust sources across all apicultural matrices investigated in this study, i.e., they appear to be minimally affected by biotransformation. To further assess the robustness and potential geographic variation of REY patterns in honey, an exploratory comparison was performed across sampling locations in different regions.

**Fig. 4:**
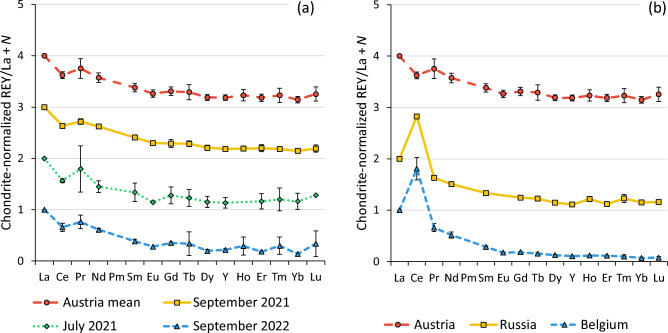
Chondrite- and La-normalized REY patterns in honey (**a**) from the same site (Austria), taken at three different occasions (September 2021: *n* = 3, July 2021: *n* = 2, September 2022: *n* = 3, mean: *n* = 8) and (**b**) from Austria (*n* = 8) compared to Russia (*n* = 2) and Belgium (*n* = 3)

Figure 4a shows the chondrite- and La-normalized REY patterns of honey samples from Austria (Leoben) collected over three different sampling campaigns (September 2021, July 2022, September 2022). The patterns overlap within the measurement uncertainties. This aligns with reports from other food matrices where REY patterns show limited intra-site temporal variability and thus utility for origin/authenticity tracing [46]. In the context of the present study, REY signatures appear temporally stable at the site level and reproducible, which indicates their potential for use as local environmental tracers.

Figure 4b compares the mean chondrite- and La-normalized REY profiles of Austrian honey with samples from Belgium and Russia. While all three patterns retain the shape typical of crustal REY profiles, the most evident deviation can be observed regarding the relative enrichment of Ce. While deeper interpretation would require complementary geochemical context (e.g., soils or water), the patterns point towards region-specific influences on REY profiles in honey, likely related to local geochemistry. However, the included Belgian and Russian honeys were not intended to provide statistically representative geographic baselines for the respective regions, as only a single sample was available from each external site. Instead, these samples serve to illustrate that REY patterns can differ across locations, thereby highlighting the potential for broader geographic variability.

To our knowledge, no study has yet systematically assessed REY patterns in honey samples from different geographical origins. The observed finding points towards the potential utility of REY patterns in this context, especially in combination with other tracers such as *δ*^87^Sr/^86^Sr. Still, the comparisons presented here are exploratory in nature and should be interpreted as hypothesis-generating rather than inferential, underscoring the need for future studies with larger sample sizes and replicated sampling sites to robustly assess geochemical or environmental drivers.

### Limitations and outlook

The scope of this study was to delineate matrix-specific elemental signatures across several apicultural materials from a single apiary and to assess their respective analytical behavior for multi-element biomonitoring. The sampling design involving three campaigns within one location was therefore optimized for cross-matrix comparison rather than for resolving fine-scale temporal dynamics. Consequently, the dataset does not constitute a structured seasonal time series, and the availability of specific matrices (e.g., pollen, propolis, wax) was governed by biological and beekeeping constraints intrinsic to operational hive management. These constraints reflect the practical conditions under which apicultural biomonitoring is typically conducted and define the intended scope of inference.

Similarly, the inclusion of two external honeys was conceptualized as an exploratory extension to demonstrate that REY patterns can differ across geographically distinct contexts. These examples were not designed to support geochemical attribution or regional fingerprinting, which would require replicated sampling across multiple hives and environmental compartments. Because paired environmental media (soils, dust, atmospheric deposition, waters) were not sampled in parallel, the PCA/HCA-derived element associations presented here represent empirical covariance structures within the apicultural matrices rather than mechanistic solubility or mobility classifications.

A further methodological limitation arises from the unavailability of matrix-matched CRMs for honey, pollen, wax, propolis, or bee bodies. While in the present study, microwave-assisted digestion and calibration were validated using a multi-element CRM of plant origin, future progress in apicultural biomonitoring would benefit substantially from the development of dedicated CRMs that enable robust interlaboratory comparability and the assessment of digestion completeness for these matrices.

Future research should incorporate structured intra-annual sampling aligned with phenology, paired environmental matrices to enable source attribution, and standardized sample preparation protocols supported by dedicated CRMs.

## Conclusion

This study provides an indication that apicultural matrices differ in their tendency to take up and reflect environmental element contents. Within the present dataset, bees and propolis emerged as the most informative biomonitors. The PCA loading vectors split the analytes into two groups. Based on covariance patterns with the matrices, one can be interpreted as particle-associated/poorly water-soluble, and the other as more soluble/bioavailable.

Propolis tended to show higher levels of elements of the group which was interpreted as particle-associated – including several TCEs – which indicates its sensitivity to atmospheric deposition processes. In contrast, honeybees were enriched in more soluble and bioavailable elements, which might be linked to both dietary uptake and external exposure during foraging. This duality suggests a complementary value of bees and propolis for assessing both particulate and dissolved pollutant pathways in terrestrial environments.

Element mass fractions generally decreased from bees to wax and honey, which underscores the biotransformation processes taking place and leading to a limited transfer of some contaminants, as reported in previous studies. In contrast, the preserved, hardly fractionated REY patterns across all matrices indicate limited biotransformation of REYs, supporting their potential utility as stable geochemical tracers.

Finally, the exploratory comparison of honey from Austria, Belgium, and Russia – though based on a small feasibility dataset – suggests that regional REY differences can potentially capture site-specific environmental signatures. Future works should include a higher number of replicate samples, ideally with replicated apiaries per region and complementary environmental media such as soil and water samples. Together, these findings underline the potential of integrated multi-matrix apicultural studies for comprehensive environmental monitoring, particularly of emerging element groups such as the TCEs.

## Experimental

All preparatory work, with the exception of microwave digestions, was performed in an ISO class 8 clean room. Ultra-pure water (18.2 MΩ cm) was obtained from a Milli-Q Element system (Merck Millipore, Germany). Nitric acid (HNO_3_, *w* = 65%, p.a. grade; Carl Roth GmbH, Germany) was purified in perfluoralkoxy-polymer (PFA) sub-boiling distillation units (DST-1000 and DST-4000, Savillex, USA). Hydrogen peroxide solution (H_2_O_2_, *w* = 30%; Merck KGaA, Germany) which was tested to assist digestion was purchased in ultra-pure quality.

All plastic labware was pre-cleaned by soaking in dilute HNO_3_ (*w* = 3%) for at least 24 h, followed by thorough rinsing with ultra-pure water and drying in a laminar flow cabinet. Weighing was performed using a BL224 BASIC analytical balance (XS instruments, Italy) with a readability of 0.01 g and a division of 0.0001 g.

### Sample collection

Sampling employed powder-free nitrile gloves and acid-cleaned polyethylene (PE) consumables. Honey was extracted with the beekeepers’ stainless-steel centrifugal extractor; all other materials (bees, pollen, wax, propolis, honeycomb) were collected directly into acid-cleaned PE containers. As no significant elevation of elements typically indicative of stainless-steel leaching (e.g., Fe, Cr, Ni) was observed in honey vs. honeycomb (wax with honey enclosed) samples, it is presumed that honey processing did not significantly alter the elemental profile of the enclosed matrix (see section *Elemental contents across apicultural matrices*).

All coordinates are given in decimal degrees (WGS 84). In Austria, polyfloral honey and all other matrix types were collected from a single location: the garden of the grammar school “Altes Gymnasium” in Leoben (47.37492° (N), 15.09211° (E)). The apiary comprised three hives located within the same school garden. As it was not possible to achieve balanced hive-specific sampling due to constraints in sample availability, all samples refer to the apiary level. Three sampling campaigns were conducted at this site:

1. 21st of September 2021: pollen (*n* = 1), bees (*n* = 6), wax (*n* = 1), honey (*n* = 1)
2. 1st of July 2022: pollen (*n* = 1), bees (*n* = 57), wax (*n* = 3), honey (*n* = 1)
3. 22nd of September 2022: propolis (*n* = 3), bees (*n* = 58), wax (*n* = 3), honeycomb (*n* = 3), honey (*n* = 3)

For all hive-related samples, care was taken to obtain representative material from across the hive. Composite samples were prepared by pooling material collected from different locations within the hive structure. Honeybee specimens were collected exclusively when found dead prior to sampling. Availability constraints linked to colony status led to a heterogeneous sample set with unequal *n* across matrices and campaigns.

Polyfloral honey was also collected at two additional locations. In Russia, one honey sample (*n* = 1) was taken in May 2022 in the village Magalinshchina (54.82° (N), 32.11° (E)). In Belgium, one honey sample (*n* = 1) was collected in June 2022 in the village Beaufays (50.57363° (N), 5.61948° (E)).

The complete list of all samples along with the analytical results can be found in the SI-2, Table A1–2.

### Calibration standards and CRMs

Calibration standard solutions were prepared gravimetrically. For the elements analyzed in standard mode (no collision/reaction gas), a 10-point calibration series was prepared using the ICP multi-element standard solution VI (Merck Certipur, Germany) and single-element standard solutions for Nb, Sb, and Ta (Inorganic Ventures, USA). For the elements analyzed with nitrous oxide (N_2_O) as reaction gas, a separate 10-point calibration series containing V, Cr, Fe, Ge, As, Se, and the REYs was prepared. This set was prepared using the custom-made multielement standard AHF-7 (Inorganic Ventures, USA), which contains the REYs in ratios close to their natural abundances, and supplemented with single-element standard solutions (Inorganic Ventures, USA).

For quality control (QC) and validation, dedicated QC solutions were prepared alongside each set of calibration standards. The CRM GBW10015 Spinach Leaves (National Research Centre for Certified Reference Materials, China) was processed in parallel with the samples to assess between-batch reproducibility. This CRM was selected for lack of more matrix-appropriate materials available, as it covers a broad range of TCEs with both certified and literature values [47]. In literature, the digestion protocol using HNO_3_ applied in this work was successfully used to digest honey [13] as well as lipid and wax matrices such as vegetable oil [48] and carnauba wax [49]. Additionally, to assess the completeness of honey digestion, an in-house reference material (RM) was prepared from D( +)-sucrose (99%, p.a., Acros organics, Belgium) spiked with the standard solutions used to prepare calibration standards specified above. Further details can be found in the SI-1, Sect. 2.

### Sample preparation

Bee samples were dried in a drying cabinet at 50 °C for approximately 3 days until constant weight was achieved. All other matrices were processed without prior drying. Microwave digestion was carried out using a Multiwave PRO closed-vessel system equipped with a 24HVT50 rotor and 30 cm^3^ polytetrafluoroethylene (PTFE)-vessels (Anton Paar, Austria).

For all samples except bees, approximately 0.2–0.5 g of sample per replicate was weighed into the digestion vessels, depending on material availability. Where sufficient sample was available, triplicates were processed. For bees, either a single individual (ca. 0.02–0.08 g, depending on size) or a pooled sample, comprising 4–5 individuals, was digested.

During initial digestion trials, a reagent mixture of 5 cm^3^ HNO_3_ (*w* = 65%) and H_2_O_2_ (*w* = 30%) was tested. However, due to excessive NO_x_ gas formation caused by the high organic content, the final protocol used 5 cm^3^ HNO_3_ (*w* = 65%) alone. In both approaches, visual inspection indicated complete digestion.

The microwave program ramped to a maximum temperature of 200 °C over 20 min and held this temperature for an additional 15 min. Maximum microwave power was 1500 W. After cooling to 70 °C, the vessels were vented and opened. Digests were transferred to pre-weighed 50 cm^3^ polypropylene (PP) tubes, and vessels were rinsed with ultrapure water to a final volume of approximately 20 cm^3^. Tubes were weighed before and after addition of the digest solution to determine the exact masses. Four procedural blanks were included with each digestion batch.

Prior to ICP-MS analysis, digests were diluted 1:10 volumetrically with ultrapure water to achieve an acidity corresponding to *w* = 2% HNO_3_. Due to the very low contents of some TCEs (e.g., REYs) in honey samples, selected samples (*n* = 21, along with 3 procedural blanks) were further concentrated by evaporation (factor of 20) on a hotplate (Analab, France) at 100 °C.

### Instrumental analysis

All measurements were performed using a PerkinElmer NexION 5000 ICP-CRC-MS/MS instrument (PerkinElmer, USA) coupled to an SC-2 DX FAST autosampler (Elemental Scientific, USA). Detailed instrumental parameters are given in the SI-1, Table S4.

After each sample, the system was rinsed with HNO_3_ (*w* = 3%). To monitor for carry-over effects, HNO_3_ blanks (*w* = 2%) matching the acid mass fractions of diluted samples and calibration standards were measured at least after every four samples. QC solutions were measured at least after every ten samples.

Indium was used as an internal normalization standard to correct for instrumental drift. Based on methods established in previous publications, isotopes at mass-to-charge ratios (*m*/*z*) affected by spectral interferences were measured with nitrous oxide (N_2_O) as reaction gas, either in on-mass or mass-shift mode (+ 16 or + 32) [47, 50].

### Data processing

Data was processed using the Syngistix software version 3.1 (PerkinElmer, USA). Limits of detection (*w*_L_ = 3 × *s*) and quantification (*w*_Q_ = 10 × *s*) were calculated based on the standard deviation *s* of the procedural blanks (*n* = 4).

To evaluate agreement between certified values and measured mass fractions in the CRM, *z*-scores were calculated according to Eq. (1) based on DIN ISO 13528:2015 (E) using the uncertainties given in the certificate.

1 $$z=\frac{{x}_{lab}-{X}_{ref}}{\widehat{\sigma }}$$

where *x*_lab_: mean result of this study, *X*_ref_: reference value, and $\widehat{\sigma }$: uncertainty of the certified value. A *z*-score ≤ 2 was considered indicative of satisfactory agreement within the uncertainty [51, 52].

The REY mass fractions were normalized to chondrite [53] and plotted following a well-established approach [54]. To avoid distortion by incomplete REY profiles, only samples in which at least 75% of the REYs were above *w*_Q_ were included in the plots. This pragmatic cut-off balances visual interpretability of the profiles with sample retention.

### Statistical analysis

Since not all matrices were available at each sampling campaign, univariate analysis was performed separately for each sampling date to ensure statistical comparability. To additionally evaluate overall elemental patterns independently of the natural abundance of individual elements, mass fractions were standardized by subtracting the overall mean per analyte and dividing by the standard deviation across all samples. The resulting dimensionless values were added to obtain *S*_all_ and *S*_REY_ as shown in Eq. (2).

2 $${S}_{j}={\Sigma }_{i=1}^{n}\frac{{w}_{ij}-{\overline{x} }_{i}}{{s}_{i}}$$

where *S*_j_: Sum parameter for sample j, *w*_ij_: Mass fraction of analyte i in sample j, *x̅*_i_: Mean mass fraction of analyte i across all samples, and *s*_i_: Standard deviation of the mass fraction of analyte i across all samples.

After checking the theoretical model assumptions, one-way repeated measures ANOVAs and Tukey’s post-hoc tests were conducted in SPSS statistics version 28 (IBM, USA), with *p*-values below 0.05 regarded as statistically significant.

PCA and HCA were performed using Python 3.11 in a JupyterLab environment. All data processing steps were implemented using the libraries pandas, numpy, scikit-learn, and matplotlib. Only elements with ≥ 50% of the results above *w*_Q_ were included in the multivariate analysis to ensure meaningful comparisons. This led to the exclusion of Be, Ge, Ag, Te, Pr, Nd, Er, Tm, Yb, Lu, Th, and U. For each sample, mean values across all procedural replicates were calculated prior to multivariate evaluation. The resulting data matrix was autoscaled (standardized as above, i.e. mean-centered and scaled to unit variance) to normalize for differences in element abundance and variance. Missing values were imputed using column-wise means (mean imputation), a simple and robust method appropriate for datasets with low rates of gaps and limited skewness. PCA was conducted using sklearn.decomposition.PCA, and the first two PCs were used for visualization (Fig. 2). HCA was performed using Ward’s linkage and Euclidean distance on the autoscaled data (scipy.cluster.hierarchy).

## Supplementary Information

Below is the link to the electronic supplementary material.

Supplementary file1 (DOCX 28 KB)

Supplementary file2 (XLSX 110 KB)

## Data Availability

The data supporting the findings of this study are openly available in the Supplementary Information.
