# Supplementary material for: Biomonitoring with bees and bee products: multielement profiles including technology-critical elements
Source: Monatsh Chem. 2026 Jan 12;157(2):193–205. doi: 10.1007/s00706-025-03425-2 (PMC12913270; doi:10.1007/s00706-025-03425-2)
Supplement: Supplementary file 1 — Supplementary file1 (DOCX 28 KB) [file 706_2025_3425_MOESM1_ESM.docx]

Supplementary Information (SI) – 1

Biomonitoring with bees and bee products: Multielement profiles including technology-critical elements

**Simone Trimmel^1^ ● Michael Schober^1^ ● Johanna A. Lube^1^ ● Thomas C. Meisel^1^ ● Thomas Prohaska^1^ ● Johanna Irrgeher^1^**

**Keywords** Chemometrics **●** Ecology **●** Mass spectrometry **●** Metals**●** Rare-earth elements

____

🖂 Johanna Irrgeher

johanna.irrgeher@unileoben.ac.at

^1^ Chair of General and Analytical Chemistry, Montanuniversität Leoben, Leoben, Austria

ORCID

Simone Trimmel: 0000-0002-2102-5392

Michael Schober: 0000-0001-6061-8185

Thomas C. Meisel: 0000-0001-5572-3212

Thomas Prohaska: 0000-0001-9367-8141

Johanna Irrgeher: 0000-0003-3192-0101

# 1. Principal component analysis (PCA)

The principal component analysis (PCA) scores and cluster assignments of the samples (mean of individual replicates) are presented in Table S1.

**Table S1** PCA scores for PC1 and PC2 and cluster assignments

| Sampling campaign | Matrix | Score PC1 | Score PC2 | Cluster |
| --- | --- | --- | --- | --- |
| September 2021 | Pollen | 3.30 | -0.13 | 2 |
| July 2022 | Pollen | 0.29 | 0.60 | 2 |
| September 2022 | Propolis | 10.65 | -7.22 | 4 |
| September 2021 | Bee | 2.57 | 5.49 | 3 |
| July 2022 | Bee | 5.19 | 4.24 | 3 |
| September 2022 | Bee | 2.26 | 5.55 | 3 |
| September 2021 | Wax | -1.51 | -1.79 | 1 |
| July 2022 | Wax | -3.25 | -0.80 | 1 |
| September 2022 | Wax | -2.98 | -1.42 | 1 |
| September 2022 | Honeycomb | -4.17 | -1.07 | 1 |
| September 2021 | Honey | -3.71 | -0.91 | 1 |
| July 2022 | Honey | -4.08 | -1.27 | 1 |
| September 2022 | Honey | -4.56 | -1.27 | 1 |

The PCA loadings and directional loading vector angles for all analytes which were included in the PCA and cluster analysis (at least 50% of all values > *w*_Q_) are shown in Table S2.

**Table S2** PCA loadings for PC1 and PC2 and directional loading vector angles

| Element | Loading PC1 | Loading PC2 | Angle deg |
| --- | --- | --- | --- |
| Li | 0.149 | -0.108 | 324.1 |
| Na | 0.080 | 0.242 | 71.7 |
| Mg | 0.108 | 0.204 | 62.0 |
| Al | 0.193 | -0.150 | 322.3 |
| Ca | 0.143 | 0.153 | 46.9 |
| V | 0.200 | -0.134 | 326.2 |
| Cr | 0.162 | -0.190 | 310.5 |
| Mn | 0.130 | 0.223 | 59.7 |
| Fe | 0.215 | -0.060 | 344.5 |
| Co | 0.159 | 0.184 | 49.2 |
| Ni | 0.135 | 0.115 | 40.4 |
| Cu | 0.111 | 0.258 | 66.8 |
| Zn | 0.130 | 0.231 | 60.6 |
| Ga | 0.144 | 0.220 | 56.8 |
| As | 0.045 | 0.146 | 73.0 |
| Se | 0.110 | 0.231 | 64.6 |
| Rb | 0.096 | 0.253 | 69.2 |
| Sr | 0.168 | 0.188 | 48.2 |
| Y | 0.198 | -0.142 | 324.3 |
| Nb | 0.184 | -0.168 | 317.6 |
| Mo | 0.133 | 0.208 | 57.5 |
| Cd | 0.098 | 0.226 | 66.6 |
| Sb | 0.206 | -0.117 | 330.4 |
| Ba | 0.173 | 0.182 | 46.5 |
| La | 0.190 | -0.135 | 324.5 |
| Ce | 0.183 | -0.168 | 317.5 |
| Sm | 0.204 | -0.125 | 328.6 |
| Eu | 0.208 | -0.111 | 331.9 |
| Gd | 0.207 | -0.116 | 330.8 |
| Tb | 0.209 | -0.106 | 333.1 |
| Dy | 0.211 | -0.106 | 333.4 |
| Ho | 0.203 | -0.084 | 337.4 |
| Ta | 0.187 | -0.053 | 344.2 |
| Tl | 0.177 | 0.152 | 40.6 |
| Pb | 0.206 | -0.057 | 344.7 |
| Bi | 0.137 | -0.093 | 326.0 |

# 2. In-house reference material (RM)

In a polypropylene (PP) tube, ICP multi-element standard solution VI (Merck Certipur, Germany), multielement standard AHF-7 (for REYs, Inorganic Ventures, USA) and single-element standard solutions for Ge, Nb, Sb and Ta (Inorganic Ventures, USA) were combined with ultrapure water in a total volume of 2 cm^3^ to yield the final mass fractions shown in Table S3 after addition of sucrose. After thorough homogenization by shaking, 8 g of D(+)-sucrose (99 %, p.a., Acros organics, Belgium) were mixed into the solution.

**Table S3** Mass fractions of the analytes in the in-house RM prepared from sucrose

| Element | *w* / ng g^-1^ |
| --- | --- |
| Li | 48 |
| Be | 490 |
| Na | 47 |
| Mg | 47 |
| Al | 47 |
| Ca | 4800 |
| V | 47 |
| Cr | 48 |
| Mn | 48 |
| Fe | 480 |
| Co | 48 |
| Ni | 48 |
| Cu | 48 |
| Zn | 480 |
| Ga | 48 |
| Ge | 95 |
| As | 470 |
| Se | 490 |
| Rb | 48 |
| Sr | 48 |
| Y | 0.34 |
| Nb | 95 |
| Mo | 48 |
| Ag | 48 |
| Cd | 48 |
| Sb | 97 |
| Te | 48 |
| Ba | 47 |
| La | 0.34 |
| Ce | 0.68 |
| Pr | 0.068 |
| Nd | 0.34 |
| Sm | 0.034 |
| Eu | 0.014 |
| Gd | 0.034 |
| Tb | 0.0068 |
| Dy | 0.034 |
| Ho | 0.0068 |
| Er | 0.014 |
| Tm | 0.0034 |
| Yb | 0.014 |
| Lu | 0.0034 |
| Ta | 1.1 |
| Tl | 48 |
| Pb | 47 |
| Bi | 47 |
| Th | 0.1 |
| U | 47 |

# 3. Instrumental analysis

The instrumental parameters for the measurements using the NexION 5000 ICP-MS/MS instrument (PerkinElmer, USA) are provided in Table S4.

**Table S4** Instrumental parameters for ICP-MS/MS multielement analysis

| Parameter | Standard mode | DRC mode |
| --- | --- | --- |
| Analytes | MVI, Nb, Sb, Ta | V, Fe, Zn, Ge, As, Se, Sb, REYs |
| Cell gas | None | N_2_O |
| Cell gas flow | - | 0.4-0.7 mL min-1 |
| Spray chamber temperature | 5 °C | 5 °C |
| Interface cones | Nickel | Nickel |
| Nebulizer | PFA-ST-40 44296 | PFA-ST-40 44296 |
| Nebulizer gas flow | 0.97-0.99 mL min-1 | 0.97-0.99 mL min-1 |
| RF power | 1600 W | 1600 W |
| Plasma gas flow | 16 L min-1 | 16 L min-1 |
| Auxiliary gas flow | 1.2 L min-1 | 1.2 L min-1 |
| Data acquisition mode | 6 sweeps/reading, 1 reading/replicate, 6 replicates | 6 sweeps/reading, 1 reading/replicate, 6 replicates |
| Dwell time per replicate | 25-150 ms | 50 ms |
| Integration time | 150-900 ms | 300 ms |
| RPa | 0-0.02 V | 0 V |
| RPq | 0.25 V | 0.45 V |
| Total time/sample | 2 min 13 s | 1 min 39 s |
| Parameter | Standard mode | DRC mode |
